# Sitagliptin is effective and safe as add-on to insulin in patients with absolute insulin deficiency: a case series

**DOI:** 10.1186/1752-1947-5-117

**Published:** 2011-03-28

**Authors:** Eiji Kutoh

**Affiliations:** 1 Biomedical Center, Tokyo, Japan; 2 Division of Diabetes and Endocrinology, Department of Internal Medicine, Gyoda General Hospital, Saitama, Japan

## Abstract

**Introduction:**

It is generally believed that incretin-based therapies are effective in patients possessing certain levels of preserved β-cell function. So far, there are no reports that show the effectiveness of dipeptidyl peptidase-4 inhibitors in patients who absolutely lack the capacity for endogenous insulin secretion.

**Case presentation:**

This report describes the efficacy of sitagliptin in three Japanese patients (a 91-year-old Japanese woman with type 1 diabetes, a 54-year-old Japanese man with type 2 diabetes and a 30-year-old Japanese man with features of both type 1 and type 2 diabetes) who had no detectable post-meal C-peptide levels. Although they were receiving intensive insulin therapy together with some oral hypoglycemic agents, their glycemic control remained poor. Sitagliptin was added to the ongoing therapeutic regimen to provide better glycemic control. Although these patients had mild hypoglycemia, effective reductions of hemoglobin A1c levels were observed without any adverse events in the liver and kidney during the following 24 weeks. Two of the patients were able to reduce their insulin doses, and one of the patients could discontinue one of the oral hypoglycemic agents. There was no weight gain or gastrointestinal complaints among the three patients. Post-meal C-peptide levels remained undetectable after sitagliptin treatment.

**Conclusion:**

This report demonstrates that sitagliptin is effective and safe as an add-on therapy to insulin in reducing blood glucose levels in patients who absolutely lack the capacity for endogenous insulin secretion. The improvement seen in glycemic control could not be due to enhanced endogenous insulin secretion, since post-meal C-peptide levels remained undetectable after sitagliptin treatment, but it could be a result of other factors (for example, suppression of glucagon levels). However, the glucagon-suppressive effect of sitagliptin is known to be rather weak and short-lived. Given this background, a novel hypothesis that the glycemic effects of this drug may be caused by mechanisms that are independent of the glucagon-like peptide 1 axis (extra-pancreatic effect) will be discussed.

## Introduction

The incretin-based drugs glucagon-like peptide 1 (GLP-1) receptor agonists and dipeptidal peptidase 4 (DPP-4) inhibitors are a new class of drugs for the treatment of type 2 diabetes [1]. GLP-1 is released from intestinal L cells in response to the ingestion of a meal and plays an important role in glucose homeostasis by stimulating glucose-dependent insulin secretion and inhibiting glucagon secretion [1-3]. Currently, two GLP-1 analogues (exenatide and liraglutide) and four DPP-4 inhibitors (sitagliptin, vildagliptin, saxagliptin and alogliptin) are on the market, and many others are under development. The latter class of drugs works by inhibiting the DPP-4 enzyme that degrades GLP-1, thereby stabilizing the intact (active) form of GLP-1. Active GLP-1 stimulates glucose-dependent insulin biosynthesis and release, and GLP-1 also suppresses glucagon release, delays gastric emptying and increases satiety. In contrast to GLP-1 analogues, DPP-4 inhibitors have no effects on gastric emptying and body weight [1-3]. Sitagliptin is the first DPP-4 inhibitor on the market. It is used as monotherapy or in combination with metformin, thiazolidinedione or sulfonylurea. It is also available in a combination product with metformin. Furthermore, its combination with insulin has recently been approved in the USA based on the large amount of clinical data [4]. Incretin-based therapies are associated with enhanced β-cell function, making them a good treatment option early in the disease when the patients still maintain sufficient levels of β-cell function [1-3]. However, it is unclear whether incretin-based drugs are still effective in patients without the capacity for endogenous insulin secretion (that is, no residual β-cell functions; for example, advanced type 2 diabetes or type 1 diabetes). Animal models have demonstrated that DPP-4 inhibitors improve glucose intolerance in early-stage diabetes, but not in the late stage of the disease [5], suggesting that DPP-4 inhibitors are more effective in the presence of functional β-cells. However, whether this is the case in humans has not been investigated. The possibility of using GLP-1 analogues in patients with type 1 diabetes is now emerging [6]. As in the animal model [5], it has been proposed that the ideal candidates for this treatment strategy are individuals with type 1 diabetes who still have significant preserved β-cell activity [6]. So far, no study has demonstrated whether DPP-4 inhibitors are also effective with those patients who absolutely lack the capacity for endogenous insulin secretion. The present report presents three cases (one with type 1 diabetes, one with type 2 diabetes and one with features of both type 1 and type 2 diabetes) where the addition of sitagliptin to the ongoing insulin therapy was considerably effective in the patients whose insulin secretory capacity (residual β-cell function) was severely diminished.

## Case presentation

Case 1 is a 91-year-old Japanese woman who had been diagnosed with acute onset of type 1 diabetes two years earlier and had been using various doses and types of insulin since that diagnosis. Her endogenous insulin secretory capacity was almost completely lost since her post-meal (approximately two hours after a meal) C-peptide levels were undetectable (below 0.1 ng/ml) and 24-hour urine C-peptide levels were below 1.1 mg/day (normal range, 20.1 to 155 mg/day). Her insulin antibodies were positive at diagnosis. Why this patient developed acute onset of autoimmune type 1 diabetes with absolute insulin deficiency is elusive.

**Figure 1:**
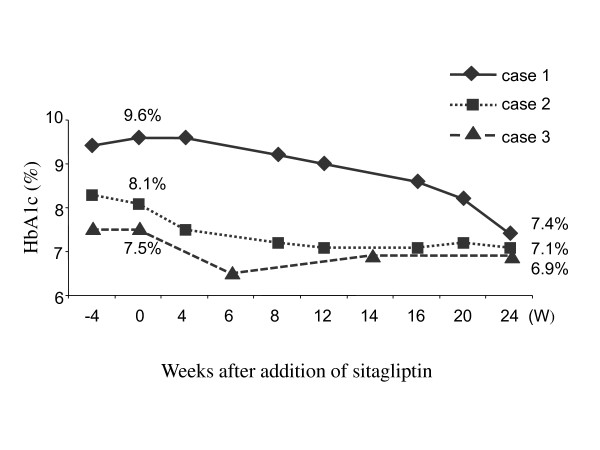
**Changes of hemoglobin A1c (HbA1c) levels after adding sitagliptin to the ongoing therapeutic regimen**. Sitagliptin was added at week 0. The changes of HbA1c levels of each patient were followed from 4 weeks prior to treatment (-4W) until 24 weeks after treatment.

This may be an atypical case. Her post-meal glucagon level was 130 pg/ml (normal range, 23 to 197 pg/ml). Her body mass index (BMI) was 21.5 (body weight/body height = 40.4 kg/137 cm). Her hemoglobin A1c (HbA1c) level was above 9.5% in the three months prior to this study. Sitagliptin (25 mg/day) was added to the ongoing therapeutic regimen to provide better control of her diabetes. At that time, she was using premixed biphasic insulin aspart (30% insulin aspart and 70% protamine-complexed insulin aspart; Novorapid 30 Mix (Novo Nordisk A/S, Bagsvaerd, Denmark) three times daily before each meal (14U, 4U and 14U, respectively). She was also taking 50 mg of miglitol three times daily and 150 mg/day aliskiren. Her post-meal blood glucose was 252 mg/dl, and her HbA1c level was 9.6% at the start of sitagliptin therapy (at baseline). She had occasionally mild hypoglycemia after starting sitagliptin, which she could manage by taking glucose drinks by herself. Her HbA1c levels were followed for 24 weeks and were reduced from 9.6% to 7.4% (2.2% reduction) (Figure 1). The doses of insulin remained unchanged, but at 20 weeks, because of the relatively good glycemic control for her age, miglitol was discontinued. Because her blood pressure started to rise at around 20 weeks, 150 mg/day aliskiren was replaced with 4 mg/day candesartan. Her post-meal C-peptide levels repeatedly remained undetectable (below 0.1 ng/ml) after sitagliptin treatment. No adverse events were observed in the kidney (assessed on the basis of blood urea nitrogen and creatinine levels) or liver (assessed by glutamate oxaloacetate transaminase, glutamate pyruvate transaminase, alkaline phosphatase and g-glutamyl traspeptidase function). No weight gain or gastrointestinal complaints were noted.

Before and during the sitagliptin treatment, no special difference in diet management or physical exercise was noted.

Case 2 is a 30-year-old Japanese man who was transferred to the emergency department with diabetic ketoacidosis and was initially diagnosed with type 1 diabetes three years prior to this study. He has been using different doses and types of insulin since that diagnosis. However, his glutamic acid decarboxylase (GAD) and insulin antibodies were negative (below detectable range). Therefore, this patient has features of both type 1 and type 2 diabetes. However, because of his clinical picture, this patient may have non-autoimmune fulminant type 1 diabetes, which is rare in Caucasian populations but comprises approximately 10% of type 1 diabetes cases in Japan [7]. His endogenous insulin secretory capacity was almost completely lost, since his post-meal C-peptide levels were repeatedly undetectable (below 0.1 ng/ml). His post-meal glucagon level was 107 pg/ml (normal range, 23 to 197 pg/ml). His BMI was 17.5 (body weight/body height = 52 kg/172 cm). His HbA1c level was above 8% in the three months prior to this study. Sitagliptin (50 mg/day) was added to the therapeutic regimen to try to achieve better control of his diabetes. At that time, he was using premixed biphasic insulin aspart (30% insulin aspart and 70% protamine-complexed insulin aspart; Novorapid 30 MIX) three times daily before each meal (15U, 7U and 16U, respectively). He was also taking 625 mg metformin three times daily and 75 mg miglitol three times daily. His post-meal blood glucose level was 242 mg/dl, and his HbA1c level was 8.1% at baseline. In the first month after starting sitagliptin, he had occasional mild hypoglycemia, which he could manage by taking glucose drinks by himself. He had no hypoglycemic events after the second month of sitagliptin therapy. His HbA1c level was followed for 24 weeks and was reduced from 8.1% to 7.1% (1.0% reduction, Figure 1). His post-meal C-peptide levels repeatedly remained undetectable (below 0.1 ng/ml) after the start of sitagliptin treatment. No adverse events were observed in kidney or liver function. No weight gain or gastrointestinal complaints were noted. At eight weeks, he could reduce the insulin dose by 2U before each meal. The doses of other drugs remained unchanged. Before and during sitagliptin treatment, no special difference in diet management or physical exercise was noted.

Case 3 is a 54-year-old Japanese man who had been diagnosed with type 2 diabetes 22 years earlier and had been using different types and doses of insulin since that diagnosis. His endogenous insulin secretory capacity was almost completely lost, since his post-meal C-peptide levels were repeatedly undetectable (below 0.1 ng/ml). His GAD and insulin antibodies were negative. His BMI was 22.9 (body weight/body height = 64 kg/167 cm). His HbA1c level was above 7.5% in the three months prior to this study. Sitagliptin (50 mg/day) was added to the patient's therapeutic regimen to try to achieve better control of his diabetes. At that time, he was using insulin lispro (Humalog, Eli Lilly and Company, Indianapolis, Indiana, USA) three times daily before each meal (8U, 10U and 10U, respectively) and insulin glargine (Lantas, Sanofi-Aventis, Paris, France) before going to bed (20U) and was also taking 2.5 mg/day rosuvastatin, 250 mg metformin three times daily, 5 mg/day amlodipine and 2 mg/day doxazosin mesylate. His post-meal blood glucose level was 221 mg/dl, and his HbA1c level was 7.5% at baseline. He had occasionally mild hypoglycemia after starting sitagliptin, which he could manage by taking glucose drinks by himself. His HbA1c level was followed for 24 weeks and was reduced from 7.5% to 6.5% (1.0% reduction) (Figure 1) at six weeks. Thus, at that time, the dose of insulin was reduced by 2U before each meal and at bedtime. The doses of other drugs remained unchanged. At 24 weeks, the patient's HbA1c level was 6.9%, which was still within an acceptable range. His post-meal C-peptide levels repeatedly remained undetectable (below 0.1 ng/ml) after sitagliptin treatment. No adverse events were observed in kidney or liver function. No weight gain or gastrointestinal complaints were noted. Before and during the sitagliptin treatment, no special difference in diet management or physical exercise was noted.

## Discussion

Physicians often encounter patients whose blood glucose control is rather difficult, even with intensive insulin therapy combined with maximally tolerated doses of oral hypoglycemic agents (OHAs). One of the features of such patients is that they lack the capacity for endogenous insulin secretion. Increasing the dosage of insulin units may be a solution to this problem; however, in practice, this can cause an increased risk of hypoglycemia and weight gain. Incretin-based drugs (GLP-1 receptor agonists and DPP-4 inhibitors) may help such patients attain desirable glycemic control. Furthermore, these drugs induce benefits in terms of post-prandial hyperglycemia control in addition to positive effects on β-cell function and possible β-cell preservation [1-3].

Recently, sitagliptin, the first DPP-4 inhibitor launched on the market, has been approved for use in combination with insulin. Sitagliptin combined with insulin significantly reduces blood glucose levels over the four-hour period following the meal test. Serum C-peptide and insulin levels were found to significantly increase over the four-hour period following the meal with sitagliptin in comparison to that in patients receiving a placebo [4]. However, few studies have demonstrated whether incretin-based drugs are still effective in patients whose insulin secretory capacities (residual β-cell function) are not preserved. Very recently, GLP-1 and its analogue were shown to be effective in C-peptide-negative patients with type 1 diabetes, though this is an off-label use of this drug [8,9]. In these reports, C-peptide remained undetectable after treatment with these agents, suggesting no improvement in β-cell function was observed. This is somewhat in contrast to the commonly accepted idea that GLP-1 augments β-cell function [1]. So far, there are no publications that have reported the effectiveness of DPP-4 inhibitors in patients who absolutely lack the capacity for endogenous insulin secretion. This report describes the results of three such patients who were given sitagliptin in addition to their ongoing intensive insulin therapy, and the glycemic control was shown to be effectively improved. Two of the patients were able to reduce their insulin doses (case 2 and case 3), and one patient was able to discontinue one of the OHAs (case 1). Interestingly, post-meal C-peptide levels remained undetectable in all three of the patients after sitagliptin treatment, suggesting that no improvement of β-cell function was observed in association with this drug. Although the limit of detection of C-peptide in the assay used in this report was 0.1 ng/ml (measured at Mitsubishi Kagaku, Tokyo, Japan), one cannot fully exclude the possibility that minimal changes in C-peptide levels in these patients may have escaped the analysis.

So, what are the potential mechanisms that can cause the reduced glucose levels with sitagliptin? A number of explanations can be postulated.

First, the glycemic effect observed in these patients may be due to suppression of glucagon levels. The mechanism by which GLP-1 suppresses glucagon secretion is far from being elucidated, and furthermore it is unclear whether endogenous insulin is a required factor for this effect. On the basis of the data from the present case report and others [8,9], one might be able to envisage that the effect of GLP-1 on glucagon secretion would not be dependent on endogenous insulin, since insulin secretion remained absent after the treatment of incretin-based drugs. Further, it was reported that the glucagon-suppressive effects of sitagliptin (or other DPP-4 inhibitors) are very small and short-lived, and also its effects on insulin secretion are somewhat not evident [10]. Therefore, it is unlikely that reduction of glucagon levels by sitagliptin can fully explain the reduced glucose levels. As a matter of fact, the postprandial glucagon levels before the addition of sitagliptin were within the normal range in the patients described in this report (cases 1 and 2). It was not possible to measure the glucagon levels after sitagliptin treatment, since glucagon has not been commercially measurable in Japan since mid-2010.

Second, the improvement seen in glycemic control with sitagliptin therapy could be due to the pharmacological effects of GLP-1, such as delayed gastric emptying and body weight loss. However, this does not seem to be the case, since in contrast to GLP-1 analogues, DPP-4 inhibitors, including sitagliptin, do not have any clear effects on these parameters [1-3]. The patients described in this case report did not have any changes in body weight or gastrointestinal complaints.

**Figure 2:**
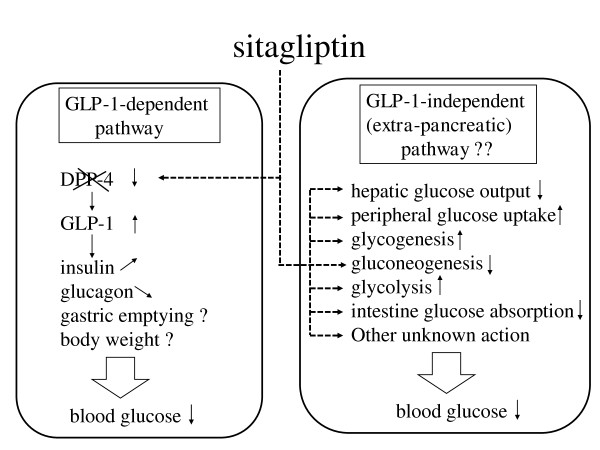
**Schematic presentation of glucagon-like peptide-1 (GLP-1)-dependent and GLP-1-independent actions of sitagliptin**.

Finally, the background information described above might suggest that the glucose-lowering effect of sitagliptin not only occurs via the GLP-1 axis but is also mediated by novel mechanisms including extra-pancreatic signal transduction pathways. Specifically, sitagliptin may have an independent role in the regulation of hepatic glucose output, glycogenesis, gluconeogenesis, glycolysis or peripheral (skeletal muscle or adipose tissues) glucose uptake different from that of insulin or glucagon action. Alternatively, it may have a novel glycemic effect, such as inhibiting glucose absorption by the intestine. These putative effects are illustrated in Figure 2. It remains to be investigated whether similar or different results will be obtained with other DPP-4 inhibitors. To further explore these issues, it is of significance to perform gene chip (microarray) analysis and identify genes regulated by sitagliptin (or other DPP-4 inhibitors), followed by cellular investigations.

The strength of this work is that similar results were obtained from patients who have very different diabetic backgrounds. But the common denominator is that these patients are absolutely insulin-deficient. A limitation of this work is that this report is based on just three observational case studies. Well-designed and adequately powered randomized clinical trials are necessary to draw well-validated conclusions.

## Conclusions

The cases presented in this report demonstrate that sitagliptin is still effective and safe in reducing blood glucose levels in those patients who have no residual β-cell function. However, one should keep in mind that sitagliptin should be used not as a replacement therapy but as an add-on to insulin with such absolutely insulin-deficient patients.

## Abbreviations

BMI: body mass index; DPP-4: dipeptidyl-peptidase-4; GAD: glutamic acid decarboxylase; GLP-1: glucagon-like peptide 1.

## Consent

Written informed consent was obtained from the patients for publication of this case report and any accompanying images. A copy of the written consent is available for review by the Editor-in-Chief of this journal.

## Competing interests

The authors declare that they have no competing interests.

## Authors' contributions

EK analyzed and interpreted the patient data and wrote the manuscript.
